# CD200R/Foxp3-mediated signalling regulates microglial activation

**DOI:** 10.1038/srep34901

**Published:** 2016-10-12

**Authors:** Min-Hee Yi, Enji Zhang, Jwa-Jin Kim, Hyunjung Baek, Nara Shin, Sena Kim, Sang Ryong Kim, Hang-Rae Kim, Sung Joong Lee, Jin Bong Park, Yonghyun Kim, O-Yu Kwon, Young Ho Lee, Sang-Ha Oh, Dong Woon Kim

**Affiliations:** 1 Department of Anatomy, Brain Research Institute, Chungnam National University School of Medicine, Daejeon, 301-747, Republic of Korea; 2 Department of Neuroscience & Cell Biology, the University of Texas Medical Branch School of Medicine, Galveston, TX 77555, USA; 3 Department of Anesthesiology, Yanbian University Hospital, Yanbian, 133000, China; 4 LES Corporation Inc., Gung-Dong 465-16, Yuseong-Gu Daejeon, 305-335, Republic of Korea; 5 Department of Plastic Surgery, Chungnam National University Hospital, Daejeon 301-721, Republic of Korea; 6 School of Life Sciences, BK21 plus KNU Creative BioResearch Group, Kyungpook National University, Daegu 41566, Republic of Korea; 7 Department of Anatomy and Cell Biology, and Biomedical Sciences Seoul National University College of Medicine, Seoul 03080, Republic of Korea; 8 Department of Neuroscience and Physiology, and Dental Research Institute, School of Dentistry, Seoul National University, Seoul 110-749, Republic of Korea; 9 Department of Physiology, Brain Research Institute, Chungnam National University School of Medicine, Daejeon 301-747, Republic of Korea; 10 Department of Chemical and Biological Engineering, The University of Alabama, Tuscaloosa, AL, USA; 11 Department of Medical Science, Chungnam National University School of Medicine Daejeon, 301-747, Republic of Korea

## Abstract

The heterogeneity of microglial functions have either beneficial or detrimental roles in specific physiological or pathological environments. However, the details of what transcriptional mechanisms induce microglia to take beneficial phenotypes remain unknown. Here, we report that Foxp3 is essential for beneficial outcome of the microglial response and depends upon signalling by the immunoglobulin CD200 through its receptor (CD200R). Foxp3 expression was up-regulated in microglia activated by excitotoxicity-induced hippocampal neuroinflammation. Suppression of CD200R prevented anti-inflammatory phenotype of microglia, but over-expression of Foxp3 enhanced it. Phosphorylation of STAT6, a downstream effector of CD200R, modulated transcription of Foxp3. Finally, CD200R/Foxp3-mediated signalling enhanced hippocampal neuronal viability and conferred a degree of neuroprotection, presumably by counteracting inducible nitric oxide synthase. We conclude that enhancement of Foxp3 through CD200R could be neuroprotective by targeting the microglia.

Microglia are resident immune cells in the central nervous system (CNS) that actively participate in both neuronal homeostasis and adult neural pathology^1^. Under normal physiological conditions, microglia survey the microenvironment and communicate bidirectionally with neurons^2^,^3^. Chronic microglial activation, however, is an important hallmark of many neurodegenerative diseases, Uncontrolled and over-activated microglia can contribute significantly to disease progression^4^. Thus, regulation of microglial activation has been the subject of intensive research efforts aiming to enhance neural repair.

Microglia in the healthy CNS are typically influenced by numerous inhibitory factors in the microenvironment, many of which are produced by neurons^5^. Neuron-microglial-cell inhibitory signalling is mediated by contact-interactions between the following factors (in neuron) and their respective receptors (in microglia): CD200 and CD200R, CD22 and CD45, CD47 and CD172a, and HSP60 and TREM2/DAP12^5^. CD200 is a highly conserved member of the immunoglobulin superfamily and is commonly expressed in cells of myeloid lineage^6^ and other cell types, including neurons^7^. Expression of the cognate CD200R, however, is limited to cells of myeloid lineage and certain populations of T cells^8^. Activated microglia and macrophages are more numerous in CD200-deficient mice after induction of autoimmune encephalitis compared with wild-type animals, suggesting that CD200-CD200R interaction plays a role in the regulation of microglial activation beneficial based^9^,^10^. Previous work in mice investigating the expression of CD200 and/or CD200R in kainic acid (KA)-induced hippocampal neurodegeneration^11^, suggests that CD200-CD200R-mediated signalling contributes to activation of microglia and may serve a beneficial function in neuroinflammation.

Regulatory T cells (Treg cells) account for 5–10% of all circulating CD4 + T cells and constitutively express the nuclear transcription factor, Foxp3. They are classically defined as cells that promote tolerance for foreign and host-derived antigens, inhibiting autoimmunity and facilitating the resolution of effector T-cell responses^12^,^13^,^14^. In the CNS, Foxp3 is up-regulated in lipopolysaccharide (LPS)-activated microglia, and microglia expressing mutant Foxp3 increase their release of inflammatory factors^15^. Although Foxp3 interacts directly with nuclear factor κB (NF-κB) and modulates its transcriptional activities, there is no histopathological evidence for Foxp3 involvement in microglial activation *in vivo*. It has been reported, however, that up-regulation of CD200 is associated with Foxp3-mediated Treg cell expansion and disease progression in acute myeloid leukemia^16^.

These and other findings previously discussed lead us to hypothesize that CD200-CD200R-mediated signalling induces expression of Foxp3 in microglia. Furthermore, the CD200R-Foxp3 pathway may be involved in beneficial function of microglia and a shift to a anti-inflammatory phenotype. Consistent with this hypothesis, we report here that Foxp3 is up-regulated in microglia and modulates activation of microglia through CD200R-mediated signaling.

## Results

### Foxp3 expression is up-regulated in activated microglia after kainic acid injection

**Figure 1:**
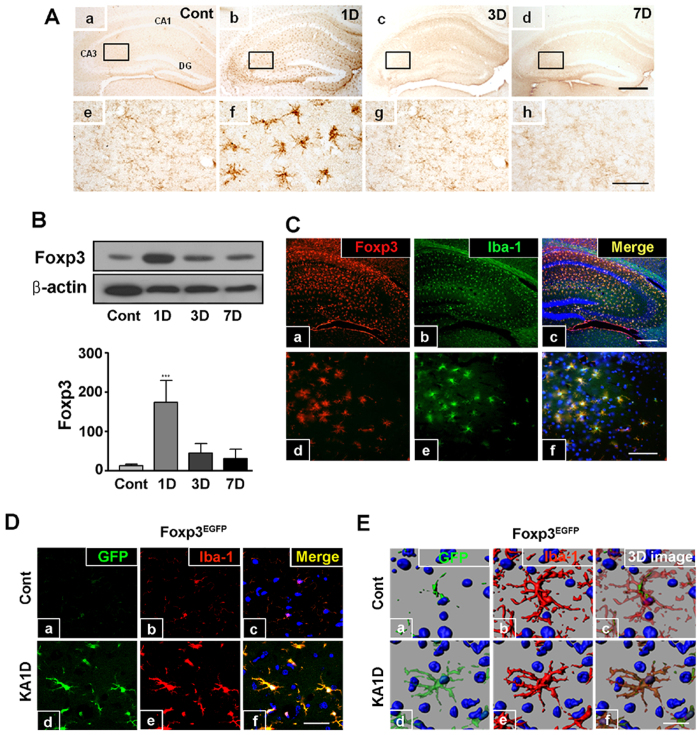
Foxp3 is up-regulated in microglia following KA-induced excitotoxicity *in vivo*. (**A**) Immunostaining for Foxp3 in mice injected (i.c.v.) with saline (a,e) or KA (b–d, f–h) 1 (b,f), 3 (c,g), or 7 (d,h) days post-injection. Higher magnification of rectangular areas (e–h) in the hippocampus showed sequential changes of Foxp3 expression. Scale bar, 100 μm (a–d) or 20 μm (e–h). (**B**) Representative immunoblotting of Foxp3 (upper panel) and relative expression of Foxp3 (lower panel). (**C**) Foxp3-positive cells (red) immunostained for Iba-1 (green) 1 day post-KA injection (yellow, merge). Scale bar, 100 μm (a–c) or 50 μm (d–f). (**D**) One day post-KA injection, Foxp3^EGFP^ mice were sacrificed and immunostained for Iba-1 (red). EGFP (green) co-localized with Iba-1 (yellow, merge) and EGFP-positive cells were more numerous in hippocampus after KA treatment. Scale bar, 2 μm. (**E**) Representative 3D confocal images showing that Foxp3-EGFP IR (green) was localized primarily in the nucleus and in some cytoplasms in controls. After KA injection, EGFP IR was observed mostly in the processes of microglia. Scale bar, 2 μm. DG, dentate gyrus. ***P < 0.001 vs. control.

To determine whether Foxp3 is involved in KA-induced hippocampal excitotoxicity, we measured Foxp3 expression in the ipsilateral hippocampus of KA-treated mice. Weak Foxp3 immunoreactivity (IR) was observed in control mice. One day after KA injection (0.1 mg/5 ml, i.c.v.), strong Foxp3 IR was observed in scattered glial cells in the ipsilateral hippocampus. Newly identified Foxp3-positive cells were observed throughout the entire hippocampus, including the CA3 region. At later timepoints, Foxp3 IR decreased and returned to control levels 7 days post-lesion (Fig. 1A). And then to eliminate the possibility of non-specific signal from the secondary antibody, isotype antibody was used (Supple. 1A). Foxp3 protein expression was confirmed by immunoblotting. Single bands on western blots corresponding to Foxp3 were detected in the hippocampi of both control and KA-injected mice. Foxp3 expression peaked at day 1, decreased at day 3, and returned to baseline levels at day 7 post-lesion (Fig. 1B).

One day after KA injection, Foxp3-positive cells exhibited a mainly pseudopodic/ameboid morphology, likely indicative of activated microglia. These observations were confirmed by double-labelling for Foxp3 and ionized calcium-binding adapter molecule (Iba-1), a known marker for microglia. Foxp3 IR was observed in Iba-1-positive microglia in KA-injected mice (Fig. 1C). No astroglial Foxp3 expression was observed at any timepoint (data not shown). To further confirm Foxp3 expression in microglia, we utilized B6.Cg-*Foxp3^tm2Tch^*/J (Foxp3^EGFP^) mice, which co-express Enhanced Green Fluorescent Protein (EGFP) and Foxp3 under the control of its endogenous promoter^17^. Weakly labelled EGFP-positive cells were detected in the hippocampi of control mice. In contrast, strong EGFP signalling was observed 1 day post-KA injection in Iba-1-positive microglia in KA-injected hippocampi (Fig. 1D). Besides Foxp3 staining was colocalized with EGFP, EGFP signal was also increased at 1 day post lesion in reporter mouse (Supple. 1C). Lastly, EGFP-positive cells were imaged in their entirety at high resolution with a confocal microscope using 3D reconstruction, and cell morphology was analyzed. In control brains, EGFP labelling was detected in resting microglia having a small cytoplasm and a few thin processes. One day after KA injection, however, EGFP-positive cells had enlarged cell bodies, thicker processes, and morphologies typical of activated microglia (Fig. 1E). These data suggest that Foxp3 is expressed in microglia in the hippocampus and that this expression is up-regulated in a pathological microenvironment, such as that induced by treatment with KA.

### Foxp3 expression is dependent upon CD200-CD200R-mediated signalling

**Figure 2:**
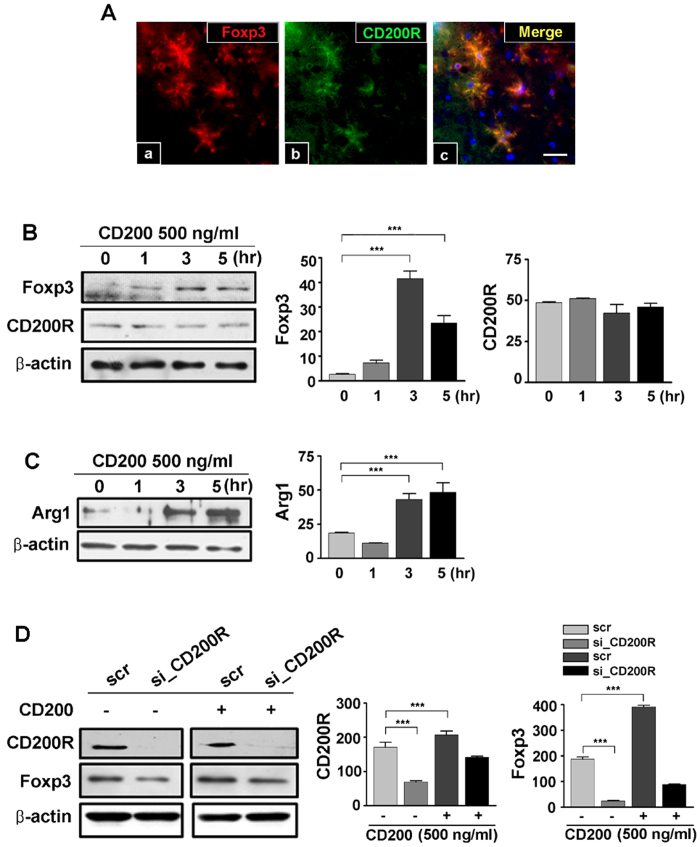
Foxp3 expression is dependent upon CD200-CD200R-mediated signalling in microglia. (**A**) One day after KA treatment, mice were sacrificed and the hippocampus was double-labelled for Foxp3 and CD200R. Foxp3 IR (red, a) co-localized with that of CD200R (green, b; yellow, merge, c). Scale bar, 20 μm. (**B**) Primary microglia were collected from neonatal rats, plated, and treated with recombinant CD200 protein for the indicated length of time (left). Foxp3 expression (middle), but not CD200R (right), increased 3 h after CD200 treatment. (**C**) Treatment of rat microglia with CD200 up-regulated expression of the beneficial microglia activation marker, Arg1. (**D**) BV2 cells were transfected with control (scr) siRNA or siRNA targeting CD200R (si_CD200R) for 24 h, followed by treatment with CD200 for 3 h. Resultant immunoblots (left) and corresponding quantification (middle, CD200R; right, Foxp3) are shown. CD200R siRNA treatment reduced Foxp3 expression, which recovered slightly with CD200 treatment. ***P < 0.001 vs. control.

The interaction between CD200 and its receptor is an important regulator of microglial activation^10^,^18^. To address whether CD200-CD200R-mediated signalling contributes to or modulates Foxp3 expression, we first double-labelled the hippocampus with CD200R and Foxp3 antibodies after treatment with KA. Foxp3 IR co-localized with that of CD200R in KA-injected mice at 1 day post-lesion (Fig. 2A). And then to eliminate the possibility of non-specific signal from the secondary antibody, isotype antibody was used (Supple. 1B). We had quantified the percentage of CD200R expression in activated microglia, Foxp3 expression in CD200R + cells and Arg1 expression in Foxp3 + cells (Supple. 1D,E). To determine whether CD200 regulates Foxp3 expression specifically in microglia and to confirm a role for CD200 in microglial polarization, we treated primary microglia with CD200, and measured Foxp3 and Arginase 1 (Arg1) expression. Treatment with CD200 stimulated Foxp3 expression in primary microglia without altering CD200R expression (Fig. 2B). Furthermore, expression of Arg1, a marker of microglia activation beneficial based, significantly increased with CD200 treatment (Fig. 2C).

To determine whether Foxp3 expression is dependent upon CD200-CD200R-mediated signalling, we employed a loss-of-function strategy utilizing CD200R short interfering RNA (siRNA, siCD200R) in cultures of BV2 microglia. Treatment with si_CD200R significantly decreased Foxp3 expression (Fig. 2D, left panel). Furthermore, expression of Foxp3 after stimulation with CD200 also decreased with si_CD200R treatment (Fig. 2D, right panel). Collectively, these data demonstrate that CD200 induces Foxp3 expression in microglia and that this expression is dependent upon CD200R signalling.

### Suppression of CD200R prevents anti-inflammatory phenotype of microglia

**Figure 3:**
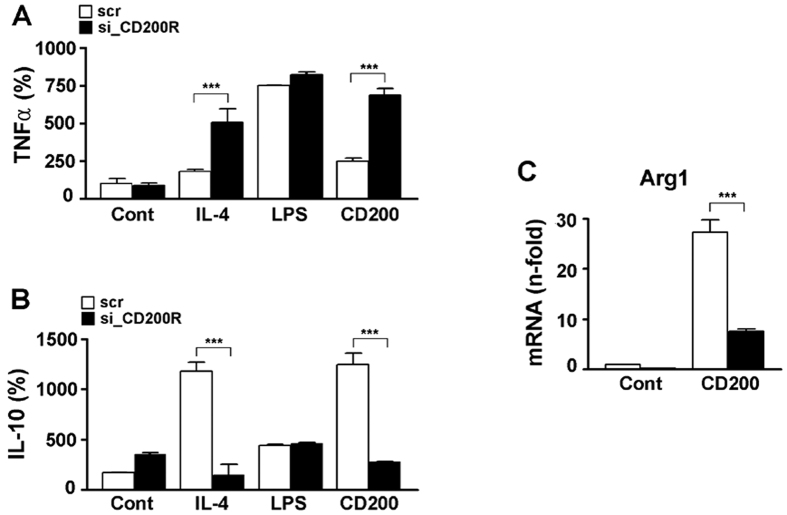
Suppression of CD200R inhibits pro-inflammation polarization of microglia and enhances microglial anti-inflammatory response. BV2 cells were incubated with scrambled RNA or si_CD200R, followed by treatment IL-4 (10 ng/ml), LPS (100 ng/ml), or CD200 (500 ng/ml) for 3 h. Supernatants were collected and assayed for levels of TNF-α (**A**) and IL-10 (**B**) by ELISA. (**C**) mRNA fold changes for *Arg1* were calculated for treatment with scr and si_CD200R under control conditions (Cont) and after treatment with CD200. After 3 h, total RNA was prepared from BV2 cells and used for quantitative real-time PCR. Relative mRNA expression levels of *Arg1* are presented as fold induction. Data are representative of three experiments. ***P < 0.001 vs. control.

Having established a role for CD200-CD200R-mediated signalling in microglial activation in cultured cells, we sought to determine whether this signalling contributes to the anti-inflammatory actions of microglia. After treatment with LPS, expression of tumour necrosis factor α (TNF-α) significantly increased in BV2 cells. Transient knock-down of CD200R with siRNA did not affect TNF-α expression, but treatment with interleukin 4 (IL-4), a strong inducer benefical function of microglia, increased expression of TNF-α, even when CD200R expression was suppressed in microglia compared with control (Fig. 3A). Similarly, treatment with CD200 also increased TNF-α levels to a degree comparable to treatment with IL-4. On the other hand, expression of IL-10, an anti-inflammatory cytokine, increased after treatment with IL-4 and CD200, but this up-regulation was attenuated when CD200R expression was suppressed (Fig. 3B).

Both IL-4 and the closely related cytokine, IL-13, signal through the IL-4 receptor (IL-4R), triggering downstream processes leading to potent anti-inflammatory activity, including up-regulation of Arg1, inhibition of NF-κB isoforms, and the production of scavenger receptors for phagocytosis^19^. Thus, we investigated Arg1 expression during CD200R suppression and found that *Arg1* mRNA levels significantly decreased with knock-down of CD200R (Fig. 3C). Collectively, these results suggest that knock-down of CD200R inhibits detrimental functions and enhances *Arg1* positive microglia beneficial based.

### Over-expression of Foxp3 enhances anti-inflammatory polarization of microglia

**Figure 4:**
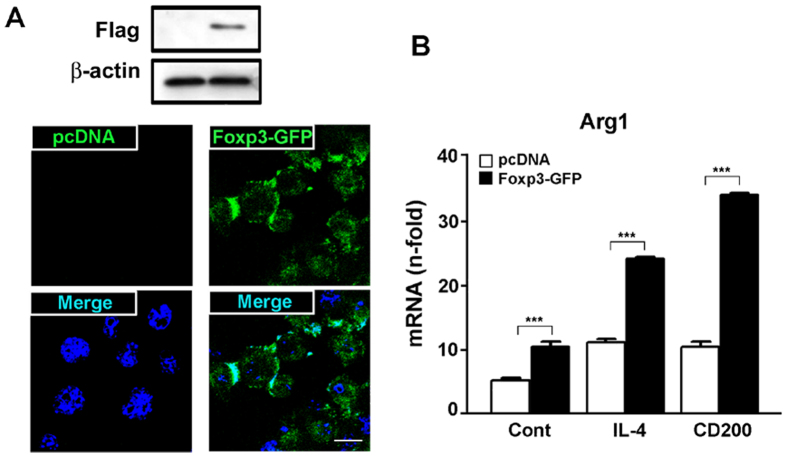
Foxp3 over-expression enhances *Arg1* positive microglial activation. (**A**) BV2 cells were transfected with mock (pcDNA) or *Flag-tagged-Foxp3-GFP* (*Foxp3-GFP*) plasmids for 24 h and Foxp3 expression was analysed by immunoblotting and immunocytochemistry with DAPI. (**B**) BV2 cells transfected with mock (pcDNA) or *Foxp3* plasmids (*Foxp3-GFP*) were cultured with IL-4 (10 ng/ml) or CD200 (500 ng/ml) for 3 h. Total RNA was prepared from BV2 cells for analysis using quantitative real-time PCR. Relative mRNA expression levels of *Arg1* are presented as fold induction. Data are representative of three experiments. ***P < 0.001 vs. control.

As activation of microglia beneficial based antagonizes the effects of detrimental function of classic miroglia activation, we investigated the effects of Foxp3 over-expression on beneficial conditions. We examined whether Foxp3 directly regulates levels of the Arg1. BV2 cells were transiently transfected with *FLAG-tagged Foxp3-GFP* (Foxp3-GFP) and stimulated with IL-4 and CD200. Arg1 expression was significantly enhanced with over-expression of Foxp3 (Fig. 4). *Arg1* mRNA levels further increased after treatment with IL-4 and CD200, suggesting that Foxp3-mediated Arg1 expression is augmented by induction of microglial activation beneficial based. Additionally, CD200 was a stronger inducer of Foxp3-mediated *Arg1* positive microglia than IL-4. These and previously presented data (Figs 2, 3, 4) demonstrate that Foxp3 is essential for activation of *Arg1* positive microglia and is dependent upon CD200-CD200R-mediated signalling.

### Phosphorylation of STAT6 regulates Foxp3 expression

**Figure 5:**
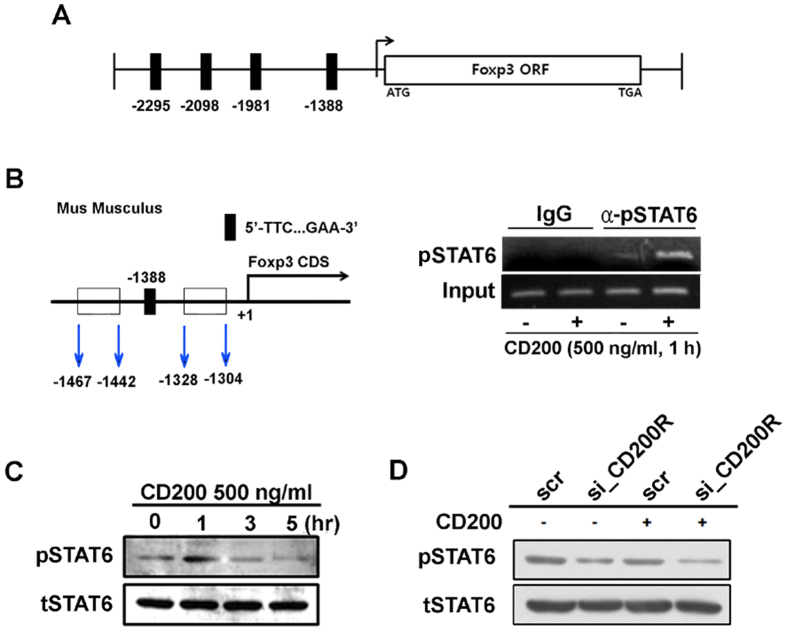
STAT6 is a downstream effector of CD200R and regulates Foxp3 expression. (**A**) A scan of mouse *Foxp3* promoters in genomic DNA identified four putative binding sites for STAT6 [TTT…GAA] within 3000 bp from the coding DNA sequence (CDS in B). (**B**) BV2 cells treated with CD200 for 1 h were subjected to a ChIP assay to evaluate STAT6 binding of the *Foxp3* promoter (right). Relative amounts of STAT6 bound to the *Foxp3* promoter were determined by PCR. PCR primers were located from −1467 to −1442 and from −1328 to −1304 (left). Total length of the amplicon was 163 bp, including the putative STAT6 binding site (−1388 to −1379, TTC…GAA). (**C**) BV2 cells were treated with recombinant CD200 protein for the indicated timeframes. Protein levels of pSTAT6 and tSTAT6 were analysed with immunoblotting. (**D**) BV2 cells were transfected with scrambled RNA or siRNA targeting CD200R (si_CD200R) for 24 h and then treated with CD200 for 1 h, followed by immunoblotting. CD200R siRNA treatment reduced phosphorylatied STAT6 (pSTAT6), which recovered slightly with treatment with CD200.

Signalling through IL-4 and IL-4R leads to phosphorylation of signal transducer and activator of transcription 6 (STAT6), which subsequently dimerizes and is trafficked to the nucleus, where it serves as a transcription factor in activated macrophages^19^. Furthermore, STAT6 can directly bind the *Foxp3* promoter^20^. Therefore, we investigated whether CD200 and IL-4 share common signalling pathways leading to microglial polarization. To verify whether *Foxp3* is a target gene for STAT6 transcriptional activity during shifting into anti-inflammatory polarization of microglia, we determined whether STAT6 directly binds the *Foxp3* promoter in our model. First, we identified potential binding sites for STAT6 (consensus site [TTT…GAA]) within the *Foxp3* promoter^21^. Sequence analysis revealed STAT6 consensus sites at −2295 bp, −2098 bp, −1981 bp, and −1388 bp upstream of the transcriptional start site of murine *Foxp3*. Next, we performed a chromatin immunoprecipitation (ChIP) assay using BV2 cells exposed to CD200. The resultant genomic DNA was analysed using ChIP-PCR assays employing primers targeting a specific region of the *Foxp3* promoter that included the putative STAT6 binding site nearest to the coding DNA sequence (CDS) (−1388 bp). A single band of the expected size (163 bp) was detected after PCR amplification (Fig. 5B), and sequencing analysis confirmed that this band contained a STAT6 binding site. No signal was detected with or without CD200 treatment when the anti-phosphorylated-STAT6 (anti-pSTAT6) antibody was substituted with IgG in the ChIP assay. Furthermore, there was an increase in the amount of pSTAT6 bound to the *Foxp3* promoter after treatment with CD200. These data suggest that STAT6 binds more readily to the *Foxp3* promoter during anti-inflammatory polarization than under control conditions.

We next assessed whether CD200-CD200R-mediated signalling is involved in phosphorylation of STAT6 in microglia. When BV2 cells were treated with CD200, phosphorylation of STAT6 increased after 1 hour (Fig. 5C), prior to the expression of *Foxp3* (Fig. 2B). We also investigated whether phosphorylation of STAT6 is dependent upon CD200R signalling. We found that treatment with si_CD200R significantly decreased phosphorylation of STAT6 (Fig. 5D). Collectively, these data demonstrate that treatment of microglia with CD200 stimulates phosphorylation of STAT6 and that this phosphorylation is required for the regulation of *Foxp3* transcriptional expression.

### CD200R/Foxp3-mediated signalling enhances viability of HT22 hippocampal neurons and suppresses nitric oxide production

**Figure 6:**
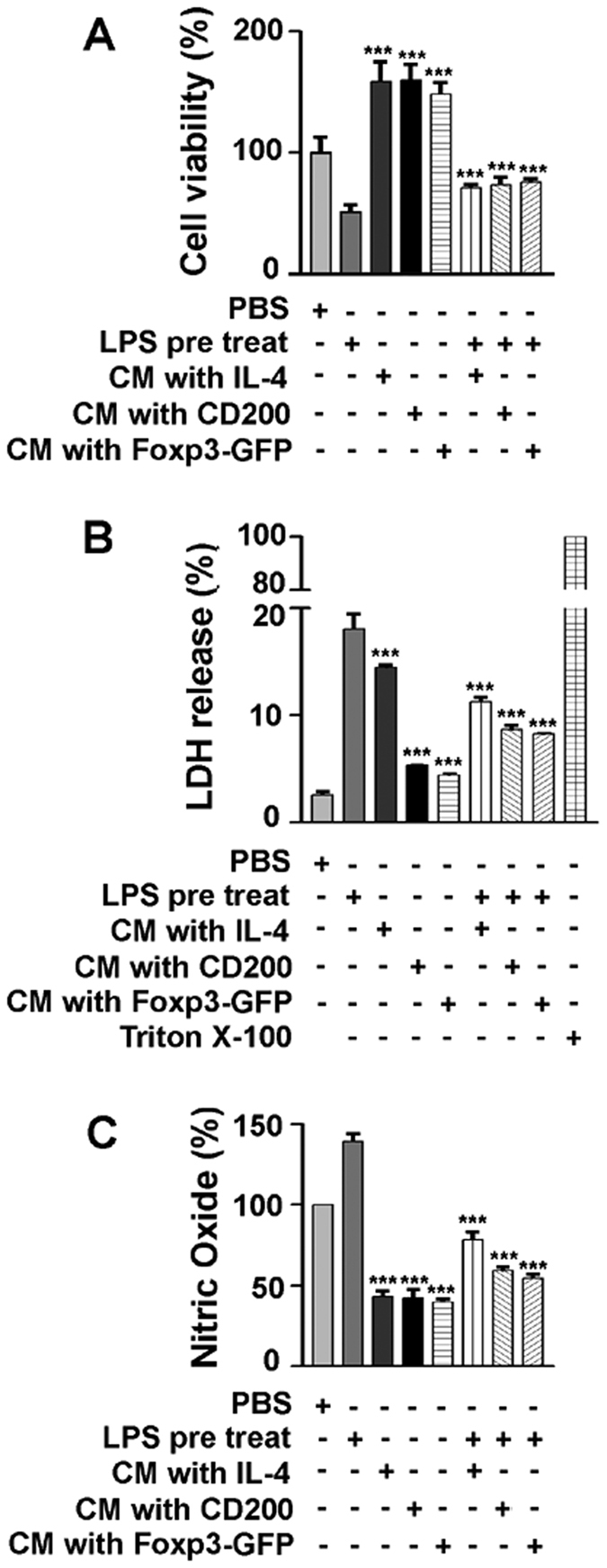
CD200R/Foxp3-mediated signalling enhances viability of HT22 hippocampal neurons and suppresses nitric oxide production. (**A**) After LPS (100 ng/ml) treatment of HT22 cells for 24 h, conditioned media (CM) from BV2 cells pre-treated with IL-4 (10 ng/ml, 3 h), CD200 (500 ng/ml, 3 h) or *Foxp3-GFP* over-expression (24 h) were added and Triton X-100 is for positive control. Cell viability of HT22 cells was analysed using a MTT assay. (**B,C**) LDH release and nitric oxide production were measured in LPS-treated HT22 cells exposed to CM. Data are representative of three experiments. ***P < 0.001 vs. control.

We sought to determine whether a transition in microglia from a shifting into anti-inflammatory polarization of microglia affects neuronal survival. We cultured HT22 hippocampal neurons using conditioned media (CM) collected from control, IL-4-treated, CD200-treated, and *Foxp3-GFP*-transfected microglial cultures. Pre-incubation of HT22 cells with LPS caused a significant decrease in neuronal cell viability, whereas pre-incubation with LPS followed by treatment with CM from cultures exposed to IL-4, CD200, or *Foxp3-GFP* effectively reversed the effects of LPS (Fig. 6A). To evaluate cytotoxicity during LPS-induced cell death in HT22 neurons, we performed lactate dehydrogenase (LDH) assays. Cell death in HT22 cells was strongly inhibited by a factor of approximately 2 after treatment with CM from cultures exposed to IL-4, CD200, or *Foxp3-GFP*, compared with pre-treatment of HT22 cells with LPS alone (Fig. 6B).

As Foxp3-positive Treg cells have been found to induce transcription of *Arg1* in bone marrow-derived dendritic cells^22^, *Arg1* may be a putative target of transcription by Foxp3. A potential role for Arg1 during inflammation is competition with inducible nitric oxide synthase (iNOS), as both Arg1 and iNOS utilize L-arginine as a substrate^23^. We sought to determine whether Foxp3 regulates the balance in activity between Arg1 and iNOS, and whether this function contributes to neuronal survival. We treated HT22 cells with LPS and then added CM collected from BV2 cells treated with IL-4, CD200, or *Foxp3-GFP*. Pre-treatment with LPS increased production of nitric oxide, but the application of CM reversed this effect (Fig. 6C). These results suggest that CD200R/Foxp3-mediated signalling in microglia confers a degree of neuroprotection in cultured neurons, potentially by counteracting iNOS.

## Discussion

**Figure 7:**
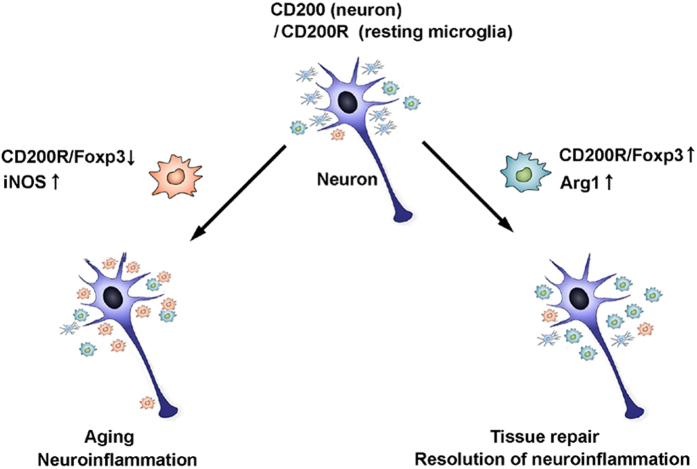
Proposed model for Foxp3 modulation of microglial polarization. Under normal physiological conditions, microglia exist in resting state, expressing CD200R, while nearby neurons express CD200. In the absence of neuronal expression of CD200, or when CD200R or Foxp3 expression in microglia is low, microglia undergo pro-inflammatory polarization, promoting neuroinflammation. When expression of CD200R or Foxp3 is elevated, anti-inflammatory polarization is enhanced, contributing to tissue repair and resolution of neuroinflammation.

In this study, we report that Foxp3 is expressed in activated microglia in the hippocampus after KA injection and that this expression is dependent upon signalling through CD200R. The underlying mechanisms appear to involve the binding of CD200 to CD200R, leading to phosphorylation of STAT6, which functions as a transcription factor for *Foxp3*, ultimately resulting in shifting into anti-inflammatory polarization of microglia. Accordingly, knock-down of CD200R inhibited microglial activation benefical based and enhanced pro-inflammatory response, while Foxp3 over-expression promoted shifting into Arg1 positive microglia. Importantly, we report that CD200R/Foxp3-mediated signalling enhanced the viability of HT22 hippocampal neurons. These data demonstrate that Foxp3 is essential for microglial activation benefical based via interaction of CD200 with its receptor, and that Foxp3 has neuroprotective effects, perhaps by promoting shifting into anti-inflammatory polarization of microglia (Fig. 7).

Foxp3 has been initially characterized as a specific intracellular marker for CD4 + and CD25 + Treg cells^24^,^25^. Although Foxp3 expression was initially characterized in Treg cells, its expression has since been reported in some non-hematopoietic normal epithelial cells, such as thymic stromal cells^26^ and breast^27^, bronchial, and prostate epithelial cells^28^. Foxp3 had been presumably found to be expressed in F4/80^hi^/CD11^int^ macrophages with immunosuppressive potential^29^, although this publication was subsequently retracted after other groups were unable to detect Foxp3 in macrophages^30^. Despite these conflicting reports, a recent study has found that Foxp3 is expressed by macrophages infiltrating mouse renal cell carcinoma tumours^31^. Its expression in other cell types remains highly controversial and is still fiercely debated. Here, we present data demonstrating the expression of Foxp3 in activated microglia in the hippocampus after treatment with KA (Fig. 1). These findings are consistent with those of a previous study reporting Foxp3 expression in microglia, which was up-regulated upon activation with LPS *in vitro*^15^.

Foxp3 has been previously thought to be exclusively found in the nuclei of CD4 + and CD25 + T cells^14^. Recently, one group has reported that the sub-cellular localization of Foxp3 can differ between subsets of Treg cells^32^, specifically in primary human CD4 + and CD25 + Treg cells and in recently activated conventional CD4 + cells. Foxp3 is expressed in the cytoplasm of human CD4 + and CD25- T cells upon activation and contains leucine-rich nuclear export signals. Our present results show that Foxp3 is expressed in the processes of activated microglia (Fig. 1A,C,D). To further characterize the sub-cellular localization of Foxp3 in microglia, we utilized Foxp3 reporter mice (*Foxp3*^*EGFP*^), wherein EGFP and Foxp3 are co-expressed under the control of the endogenous *Foxp3* promoter^17^. Three-dimensional reconstruction using confocal microscopy revealed that Foxp3 is expressed in the cytoplasm of resting microglia. This expression was up-regulated in activated microglia in both the cytoplasm and nucleus (Fig. 1E). This conditional expression pattern of Foxp3 may provide insight on the role of Foxp3 in microglia: Foxp3 is a potentially critical factor in microglial polarization and in determining activational phenotype. It is clear from our results that Foxp3 is expressed in microglia under normal physiological conditions, and its expression is up-regulated in activated microglia after exposure to inflammatory stimuli.

CD200 is expressed in various cell types, including neurons^10^ and astrocytes^33^, whereas CD200R is expressed primarily by cells of myeloid lineage and by microglia in the brain^6^,^34^. It is well established that the interaction between CD200 and its receptor is an important factor in modulating microglial activation and that loss of CD200 is accompanied by an increase in microglial activation^10^,^18^. CD200 deficiency has also been associated with an enhanced responsiveness of microglia to stimulation by toll-like receptor agonists, LPS, Pam_3_CSK_4_^33^, and interferon-γ^35^. CD200R has been confirmed to be expressed in activated microglia beneficial based in the hippocampi of KA-treated mice^11^. Corroborating these findings, we show here that Foxp3 is expressed by activated microglia in the hippocampi of KA-treated mice and that it is co-expressed with CD200R. Collectively, our results and those of others suggest that Foxp3 expression is dependent upon CD200R signalling and serves to enhance *Arg1* positive microglial polarization (Figs 2, 3, 4).

CD200R/Foxp3-mediated microglial activation is similar to immunosuppressive type 2 differentiation of macrophages in tumour microenvironments. Myeloid cells are important cells in the tumour microenvironment, capable of regulating immune response and potentiating tumour growth. Cancer stem cells can evade the immune system by generating a tolerogenic response facilitated by the immunosuppressive factor, CD200^7^. As Foxp3 is expressed in macrophages infiltrating mouse renal cell carcinoma tumours^31^, it is reasonable to hypothesize that CD200-expressing tumour cells may interact with CD200R-expressing macrophages. This interaction could thereby trigger CD200R-mediated Foxp3 expression, resulting in Foxp3-positive, type 2-differentiated macrophages with immunosuppressive effects in different tumour microenvironments. Consistent with this hypothesis, CD200-expressing tumour cells inhibit CD200R-expressing tumour-associated macrophages, resulting in decreased tumour growth^7^.

STAT6, a member of the Signal Transducer and Activator of Transcription family of proteins, is primarily activated by two cytokines, IL-4 and IL-13^36^. The downstream effects of IL-4 are mediated by IL-4 receptor alpha (IL-4Rα). Upon binding its ligand, IL-4Rα dimerizes, either with the common γ-chain to produce a type 1 signalling complex, primarily found in hematopoietic cells, or with interleukin-13 receptor alpha 1 (IL-13Rα1) to produce a type 2 complex, which is also expressed in non-hematopoietic cells^37^,^38^. The type 1 signalling complex is critical for Th2 skewing of T cells and the differentiation of alternatively-activated macrophages, whereas the type 2 complex plays a role in the non-hematopoietic response to IL-4 and IL-13, for example, in airway hyper-reactivity and mucous production^19^. Upon activation, the type 1 complex signals through Janus family kinases (JAK1 and JAK3), which phosphorylate and create docking sites for the transcription factor, STAT6, which then dimerizes and translocates to the cell nucleus. STAT6 promotes IgE class switch (in B cells) and the transcription of various factors, including GATA3 (a Th2 cell inducer), brain-derived neurotrophic factor and nerve growth factor (in astrocytes), and major histocompatibility complex II (MHCII), Ym-1, and Arg1 (in myeloid lineage cells)^19^. In macrophages, STAT6 promotes IL-4-induced differentiation of alternatively-activated macrophages and is associated with suppression of T-cell proliferation^39^. To the best of our knowledge, this is the first to report that phosphorylation and the binding of STAT6 to the *Foxp3* promoter are enhanced by the interaction between CD200 and CD200R in microglia.

Previous studies support the idea of an opposing relationship between STAT6 and Foxp3 in Treg cells. Dardalhon *et al*. show that IL-4 inhibits tumour growth factor (TGF)-β-induced expression of Foxp3 in naïve T cells *in vitro* via a STAT6-dependent mechanism^40^. Takaki *et al*. demonstrate that STAT6 can directly bind to the *Foxp3* promoter and suppress TGF-β-dependent induction of Foxp3 expression^20^. Additionally, *STAT6 (−/−)* mice are highly resistant to Th2-driven lung inflammation, in part due to the augmentation of Treg cell populations, suggesting that STAT6 suppresses Treg cell proliferation *in vivo*^41^. Involvement of STAT6 in the differentiation and survival of Treg cells has also been investigated. The absence of STAT6 impairs the generation of Ag-specific CD4 + /CD25 + /Foxp3-positive cells. Moreover, in transgenic mice expressing a constitutively active form of STAT6, the fraction of CD4 + and Foxp3-positive cells exceeds that of control wild-type littermates^42^. Our data demonstrate that STAT6 binds directly to the *Foxp3* promoter and up-regulates Foxp3 expression during CD200-induced activation of *Arg1* positive microglia beneficial based (Figs 2 and 5).

Microglia maintain a healthy microenvironment required for neuronal function. In particular, M2a microglia contribute to cellular repair and regeneration through the production of anti-inflammatory and immune-regulatory factors. M2b/c cells constitute the deactivating phenotype and also express anti-inflammatory factors^43^. Consequently, there remain unanswered questions regarding the effects of M2 microglial activation during neuroinflammation. We thus investigated the viability of HT22 hippocampal neurons during increased CD200R/Foxp3-mediated signalling. HT22 cell viability was enhanced under conditions promoting microglial activation beneficial based (Fig. 6). These results may provide insight on the development or progression of neurological disorders associated with neuroinflammation.

In summary, our findings demonstrate that Foxp3 expression is up-regulated in microglia following KA-induced excitotoxicity. Expression of Foxp3 is dependent upon the interaction between CD200 and its receptor, CD200R, and regulates beneficial activation of microglia via CD200R- and STAT6-mediated signalling. Finally, CD200R/Foxp3-mediated signalling confers neuroprotective effects, possibly by promoting anti-inflammatory polarization of microglia.

## Materials and Methods

### Antibodies and regents

All commercial antibodies and reagents were purchased from the following sources: anti-Foxp3 (ab22510, 1:200 (IHC, IF), 1:1000 (WB) dilution) antibody was from Abcam; anti-actin (A5316, 1:5,000 dilution), anti-Flag (F7425, 1:5000 dilution) antibody was from Sigma-Aldrich; anti-Iba-1 (019–19741, 1:400 dilution) antibody was from Wako; anti-CD200R (AF2554, 1:500 dilution) antibody was from R&D Systems; Hoechst (33342, 1:1000 dilution) antibody was from Invitrogen; anti-Arg1 (sc-20150, 1:1000, dilution), anti-phopho-STAT6 (sc-11762, 1:1000 dilution), anti-STAT6 (sc-621, 1:1000 dilution) antibodies were from Santa Cruz Biotechnology; CD200 recombinant protein (2554-CD, 500 ng/ml) were from R&D Systems; and bacterial LPS were from Sigma-Aldrich.

### Experimental animals and lesions

Male imprinting control region (ICR) mice (Samtako, Osan, Rep. of Korea) weighing 23–25 g were used in this study. B6.Cg-*Foxp3*^*tm2Tch*^/J (Foxp3^EGFP^) was purchased from the Jackson Laboratory. Animals were housed in a room under a controlled light/dark cycle (12 h light/12 h dark) at 23 °C. Food and water were available *ad libitum*. All experiments were carried out with the approval oc the Animal Care and Use Committee at Chungnam National University (CNU-00151) and were consistent with the ethical guidelines of the National Institutes of Health and the International Association. KA (Sigma, USA) was prepared as a stock solution at 5 mg/ml in sterile 0.1 M phosphate-buffered saline (PBS; pH 7.4); aliquots were stored at −20 °C until required. Injection of KA (0.1 mg/5 ml in PBS, i.c.v.) was performed according to a previously established procedure^44^,^45^. Briefly, KA was injected at bregma using a 50 μl Hamilton microsyringe fitted with a 26-gauge needle inserted to a depth of 2.4 mm. Control mice received an equal volume of saline. KA-injected animals (n = 8/group) and saline-injected control animals (n = 6) were allocated to four groups, which were sacrificed at predetermined times after KA administration.

### Immunohistochemistry and Confocal imaging analysis

Parallel free-floating sections were subjected to endogenouse peroxidase blocking with 1% H_2_O_2_ in PBS, followed by treatment with blocking buffer 1% fetal bovine serum (FBS) in PBS and 0.3% Triton X-100 for 30 min and incubation with primary anti-Foxp3 antibody. Immounohistochemical staining of the tissue sections was performed using the avidin-biotin peroxidase complex (ABC) method as previously described^44^,^45^. In order to simultaneously demonstrate a pair of antigens, anti-Foxp3 was stained with one of the following antibodies in the same section: Iba1, CD200R. Cy^3^-or Cy^2^-conjugated secondary antibody was used. All immunoreactions were observed under Axiophot microscope (Carl Zeiss, Germnay) and confocal laser-scanning microscope (LSM 700; Zeiss, Thomwood, NY USA).

### Microglia Cell culture

For primary microglia culture, 1 day postnatal SD rat pup (Samtako, Osan, Rep. of Korea) was decapitated in an ice-chilled dish, and then, the brain was removed. After removing the meninges, the cerebral cortex was dissected and dissociated in Hank’s balanced salt solution (HBSS) with 5.5 mM glucose, 20.4 mM sucrose, and 4.2 mM sodium bicarbonate. After centrifugation, the cells were plated on poly-L-lysine–coated T75 flasks and maintained in minimum essential medium (MEM) containing 20% FBS, 100 μm non-essential amino acid solution, 2 mM L-glutamine, and antibiotics. After 7 days, the flasks were agitated on an orbital shaker for 2 h at 200 rpm at 37 °C, and supernatant with floating cells was collected. After seeding, cells were allowed to incubate for 1 h to enable microglia to attach to the dish, and then washed once with culture media to remove non-adherent cells (astrocytes). Microglial cells were counted and seeded in MEM growth media containing 10% FBS, 100 μM nonessential amino acid solution, 1.4 mM L-glutamine, and antibiotics. Under these conditions, the purity of the microglial population was 95%, as determined by immunofluorescence analysis using anti-iba-1 to detect microglial cells, anti–2′, 3′-cyclic nucleotide 3′-phosphodiesterase (CNPase) to detect oligodendrocytes, and glial fibrillary acidic protein to identify astrocytes. The murine microglia cell line BV2 and hippocampal neuronal cell line HT22 were maintained in DMEM/F12 (PAA) supplemented with 5% heat inactivated FBS and antibiotics. All cultures were kept at 37 °C in 5% CO_2_ humidified air atmosphere. Prior to treatment cells were washed with PBS and serum-free medium was added. After stabilization, cells were treated with 100 ng/ml LPS from *Escherichia coli* 026:B6, 10 ng/ml recombinant rat IL-4 and 500 ng/ml recombinant CD200.

### Transfection with Small Interference RNA

For transfection with small interference RNA (siRNA), BV2 cell were trypsinized and seeded in a 60-mm dish at 60% confluence. si_CD200R (sense sequence: CCAAUUAUUAGGAUCAUACAUUCAA, NM_023953) was purchased from Integrated DNA Technologies, Inc. The individual siRNAs, oligofectamine, and Opti-MEM (Invitrogen, MD) were mixed and incubated at room temperature for 20 min. siRNA-oligofectamine complexes were incubated with the cells for 5 h in Opti-MEM with 10% FBS (siRNA at 20 nM final concentration). siRNA oligofectamine complexes were removed and the cells were placed in growth media for 24 h. At 3 h after CD200 recombinant protein treatment at the indicated concentrations, the cells were processed for immunoblotting, ELISA (R&D system, #558534 and #555252) and real-time PCR.

### DNA transfection

For overexpression of *FLAG-tagged-Foxp3-GFP* (FLAG-Foxp3) (a kind gift from Sin-hyeog Im, GIST, Gwangju, Rep. of Korea), transfection in BV2 cells was carried out using a WelFect EXTM Plus (WELGENE, #TR 003-02, Rep. of Korea) according to the manufacturer’s instructions. Transfection efficiency was more than 70%, compared to control transfection of pcDNA3.1 MOCK.

### MTT assay

To determine cell viability, assay was carried out using the 3-(4,5-dimethylthiazol-2-yl)-2,5-diphenyltretrazolium bromide (MTT, Thiazolyl blue, Carlroth, #4022.2) colorimetric assay, based on the reduction of tetrazolium salt. The HT22 cells were inoculated in a 96-well (1 × 10^5^ cells/well) and treated with conditioned media for 24 h. After incubation, 10 μl of the MTT working solution (5 mg/mL in PBS) was added to each well and incubated at 37 °C for 4 h. After removing the media, formazans into the cells were dissolved with 150 μl dimethyl sulfoxide (DMSO). Absorbance at 540 nm was measured using a microplate reader (OpsysMR, DYNEX technologies, #1MRA-2067), and cell viability was determined as the percentage of MTT reduction, assuming the absorbance of control cells as 100%.

### LDH measurement

To determine cell death, assay was quantified by the measurement of the cytosolic enzyme lactate dehydrogenase (LDH) using the Cytotoxicity Detection kit, LDH (Roche Molecular Biochemicals, Mannheim, Germany). The HT22 cells were inoculated in a 96-well (1 × 10^5^ cells/well) and treated with conditioned media for 24 h. After incubation, the supernatant was transferred to a fresh plate, and mixture (100 μl) of dye solution and catalyst was added to each well to the supernatant. Absorbance at 490 nm was measured using a microplate reader, and cell death was determined as the percentage of total LDH release, assuming the absorbance of control cells as 100%.

### Nitric oxide concentration assay

The HT22 cells were inoculated in a 96-well (1 × 10^5^ cells/well) and treated with conditioned media 24 h. After incubation, the presence of nitrite, a stable oxidized product of NO, was determined in cell culture media by Griess reagent (Sigma, #G4410, USA). Briefly, 100 μl of culture supernatant was removed and combined with 100 μl Griess reagent in a 96-well plate, followed by spectrophotometric measurement at 540 nm.

### Immunoblot analysis

Hippocampi from KA-treated (1, 3 and 7 days) and control mice were dissected and homogenized in lysis buffer (PRO-PREP reagent, Intron Biotechnology, Sungnam, Rep. of Korea). Cultured primary microglia, BV-2, and HT22 cells were collected by scraping, and the pellet was solubilized in lysis buffer (1X RIPA buffer, #9806, Cell Signaling). After centrifugation, protein concentrations were determined in supernatants using MicroBCA protein assay kits; bovine serum albumin was used as standard (Pierce Chemical, USA). Aliquots containing 30 μg protein were resolved by 10% sodium dodecyl sulfate polyacrylamide gel electrophoresis (SDS-PAGE) and visualized by enhanced chemiluminescence, according to the manufacturer’s instruction (Amersham).

### Quantitative real-time PCR analysis

RNA was isolated from BV2 with TRIzol (Invitrogen Corporation, Carlsbad, CA, USA), according to the manufacturer’s instructions. RNA concentration and purity were assessed using a NanoDrop spectrophotometer (NanoDrop Technologies, Inc., Wilmington, DE, USA). cDNA was synthesized using 1 mg of total RNA and QuantiTect Reverse Transcription Kit (Qiagen, CA, USA). PCR amplification was performed using cDNA, Power SYBR Green PCR Master Mix (Applied Biosystems, CA, USA) with following primer (Arg1, Forward: 5′-TCA TGG AAG TGA ACC CAA CTC TTC, Reverse: 5′-TCA GTC CCT GGC TTA TGG TTA CC; GAPDH, Forward: 5′-ATG ACT CCA CTC ACG GCA AAT TC, Reverse: 5′-TGG GGT CTC GCT CCT GGA AGA TG). Amplification reactions were performed in triplicate with a StepOne Plus system (Applied Biosystems, CA, USA). The threshold cycle (Ct) of the GAPDH gene was used as a reference control to normalize the expression level of the target gene (ΔCt) to correct for experimental variation. The relative level of gene expression (ΔΔCt) was calculated as ΔCtCD200-treated group -ΔCtNon-treated control, and the relative fold changes were determined by using the 2-ΔΔCt method^46^. Statistical analysis of the difference in gene expression (2-ΔΔCt values) levels between CD200-treated group and non-treated controls was calculated by a nonparametric Mann-Whitney U test (SPSS ver 20.0). A *p*-value < 0.05 was considered to indicate statistical significance.

### Chromatin Immunoprecipitation (ChIP) assay

For ChIP assay, chromatin immunoprecipitation assay kit (Upstate Biotechnology, Lake Placid, NY) was used following the manufacturer’s instructions. Briefly, BV2 cells were activated with LPS, IL-4 and CD200 or left unstimulated. After 1 h, cells were fixed with 1% formaldehyde for 10 min, washed, and harvested in SDS lysis buffer. After sonication, lysates containing soluble chromatin were incubated overnight with an anti-Foxp3 antibody or with normal mouse IgG. DNA–protein immunocomplexes were precipitated with protein A-agarose beads, washed, and eluted. An overnight incubation with sodium chloride (final concentration of 0.2 M) at 65 °C was performed to reverse cross-link DNA–protein complexes. The samples were proteinase K-digested as recommended, purified with phenol/chloroform, and precipitated with ethanol. Elutes were used as templates in PCRs using the primers (Forward: 5′-CAA CTC TGA TAA GCC CCA GAC ATG A Reverse: 5′-CCT GCT GTG TTT GTG TGT GCA TGT).

### Statistical procedures

The data were expressed as means ± standard error (SE). Statistical significance between multiple groups was compared by one-way analysis of variance (ANOVA) followed by an appropriate multiple comparison test. Two group analysis was performed using the Student’s *t*-test. *P*-values < 0.05 were considered statistically significant. All statistical analyses were performed using GraphPad Prism4 software (GraphPad Software Inc.).

## Additional Information

**How to cite this article**: Yi, M.-H. *et al*. CD200R/Foxp3-mediated signalling regulates microglial activation. *Sci. Rep*. **6**, 34901; doi: 10.1038/srep34901 (2016).

## Supplementary Material

Supplementary Information
