# Author Correction: CD200R/Foxp3-mediated signalling regulates microglial activation

**DOI:** 10.1038/s41598-020-62310-6

**Published:** 2020-03-23

**Authors:** Min-Hee Yi, Enji Zhang, Jwa-Jin Kim, Hyunjung Baek, Nara Shin, Sena Kim, Sang Ryong Kim, Hang-Rae Kim, Sung Joong Lee, Jin Bong Park, Yonghyun Kim, O-Yu Kwon, Young Ho Lee, Sang-Ha Oh, Dong Woon Kim

**Affiliations:** 1 Department of Anatomy, Brain Research Institute, Chungnam National University School of Medicine, Daejeon, 301-747 Republic of Korea; 2 Department of Neuroscience & Cell Biology, the University of Texas Medical Branch School of Medicine, Galveston, TX 77555 USA; 3 Department of Anesthesiology, Yanbian University Hospital, Yanbian, 133000 China; 4 LES Corporation Inc., Gung-Dong 465-16, Yuseong-Gu Daejeon, 305-335 Republic of Korea; 5 Department of Plastic Surgery, Chungnam National University Hospital, Daejeon, 301-721 Republic of Korea; 6 School of Life Sciences, BK21 plus KNU Creative BioResearch Group, Kyungpook National University, Daegu, 41566 Republic of Korea; 7 Department of Anatomy and Cell Biology, and Biomedical Sciences Seoul National University College of Medicine, Seoul, 03080 Republic of Korea; 8 Department of Neuroscience and Physiology, and Dental Research Institute, School of Dentistry, Seoul National University, Seoul, 110-749 Republic of Korea; 9 Department of Physiology, Brain Research Institute, Chungnam National University School of Medicine, Daejeon, 301-747 Republic of Korea; 10 Department of Chemical and Biological Engineering, The University of Alabama, Tuscaloosa, AL USA; 11 Department of Medical Science, Chungnam National University School of Medicine, Daejeon, 301-747 Republic of Korea

Correction to: *Scientific Reports* 10.1038/srep34901, published online 12 October 2016

This Article contains errors in both the PDF and HTML versions. An affiliation was omitted for Jwa-Jin Kim. The correct affiliations for Jwa-Jin Kim are listed below:

Department of Anatomy, Brain Research Institute, Chungnam National University School of Medicine, Daejeon, 301-747, Republic of Korea.

LES Corporation Inc., Gung-Dong 465-16, Yuseong-Gu Daejeon, 305-335, Republic of Korea.

In addition, in Figure 1, panel A-g is a duplicate of panel A-e. The correct panel A-g appears below.

**Figure 1:**
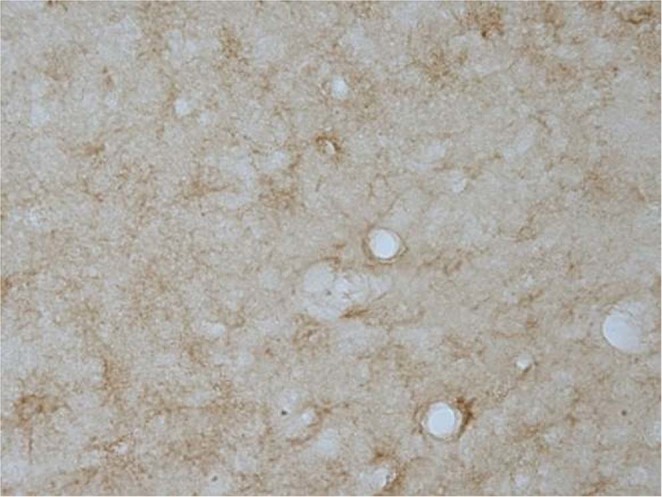
.

