# Supplementary material for: CD200R/Foxp3-mediated signalling regulates microglial activation
Source: Sci Rep. 2016 Oct 12;6:34901. doi: 10.1038/srep34901 (PMC5059636; doi:10.1038/srep34901)
Supplement: Supplementary Information [file srep34901-s1.doc]

**CD200R/Foxp3-mediated signalling regulates microglial activation**

Min-Hee Yi, Enji Zhang, Jwa-Jin Kim, Hyunjung Baek, Nara Shin, Sena Kim, Sang Ryong Kim, [Hang-Rae Kim](http://www.sciencedirect.com/science/article/pii/S0171298514000540), Sung Joong Lee, Jin Bong Park, Yonghyun Kim, O-Yu Kwon, Young Ho Lee, Sang-Ha Oh, Dong Woon Kim

**Supplementary figure legends**

**Supple. 1. Isotype control of Foxp3 and CD200R is not stained in KA-induced excitotoxic lesion in vivo and CD200R/Foxp3 is involved for regulation microglia activation** (A) Immunostaining by using isotype antibody of Foxp3 in mice injected (i.c.v.) with saline (Cont) or KA 1 day post-injection. (B) Immunostaining by using isotype antibody of CD200R in mice injected (i.c.v.) with saline (Cont) or KA 1 day post-injection. (C) Foxp3-positive cells (red) immunostained for GFP (green) 1 day post-KA injection (yellow, merge). (D) Immunostaining for CD200R expression in Iba-1+ cells, Foxp3 expression in CD200R+ cells and Arg1 expression in Foxp3+ cells in mice injected (i.c.v.) with KA 1 day post-injection. (E) Quantification for CD200R expression in Iba-1+ cells, Foxp3 expression in CD200R+ cells and Arg1 expression in Foxp3+ cells in mice injected (i.c.v.) with saline (Cont) or KA 1 day post-injection.

**
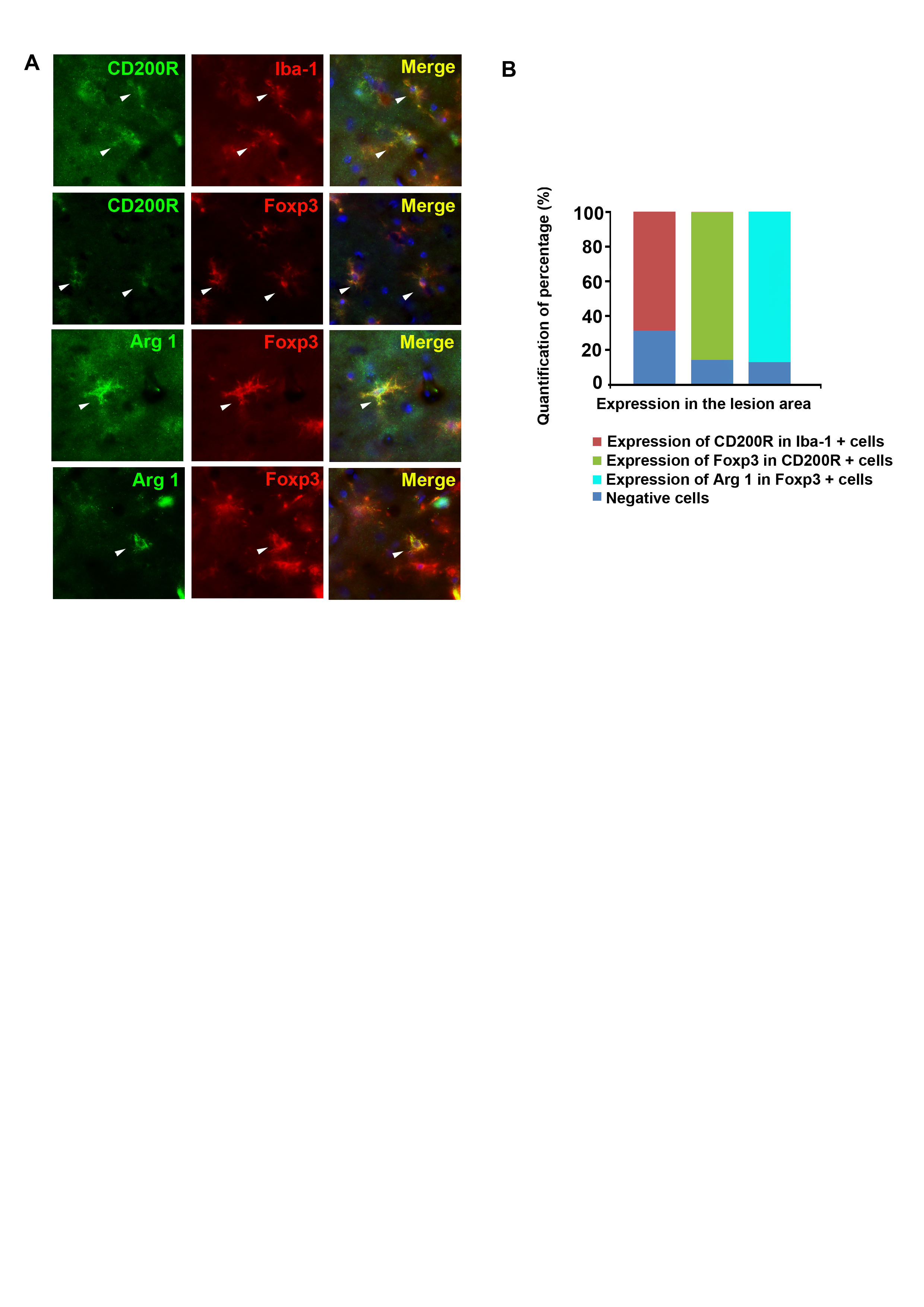
**
